# Supraventricular Runs in 7-Day Holter Monitoring Are Related to Increased Incidence of Atrial Fibrillation in a 3-Year Follow-Up of Cryptogenic Stroke Patients Free from Arrhythmia in a 24 h-Holter

**DOI:** 10.3390/jcdd8070081

**Published:** 2021-07-19

**Authors:** Andrzej Kułach, Milena Dewerenda, Michał Majewski, Anetta Lasek-Bal, Zbigniew Gąsior

**Affiliations:** 1 Department of Cardiology, Medical University of Silesia, 40-635 Katowice, Poland; zgasior@sum.edu.pl; 2 Upper-Silesian Medical Center, Department of Neurology, 40-635 Katowice, Poland; milena.dewerenda@gmail.com; 3 Upper-Silesian Medical Center, Department of Cardiology, 40-635 Katowice, Poland; mic.majewski@gmail.com; 4 Department of Neurology, Medical University of Silesia, 40-635 Katowice, Poland; abal@sum.edu.pl

**Keywords:** atrial fibrillation, cryptogenic ischemic stroke, Holter ECG, supraventricular arrhythmia, SV runs

## Abstract

Introduction: Silent atrial fibrillation (AF) is a common cause of cryptogenic ischemic stroke (CIS). The 24-h-Holter is insufficient to reveal an occult arrhythmic cause of stroke and the strategy to select the patients for long-term monitoring is missing. Objectives: The aim of the study was to evaluate 7-day-Holter monitoring to identify cases with the arrhythmic cause of stroke in CIS patients in whom 24-h-Holter was free from arrhythmia, and to assess the relation between supraventricular (SV) runs in baseline Holter and the incidence of AF in a 3-year follow-up period. Methods: 78 patients (aged 60 ± 9 years, 45 males) with CIS and no arrhythmic findings in 24-h-Holter were enrolled. All patients had 7-day-Holter monitoring after stroke and were followed up for 36 months, and then 7-day Holter was repeated. We assessed SV runs (≥5 QRS) in the initial 7-day Holter and analyzed the relation of the findings with clinical characteristics of novel AF episodes revealed early after stroke and during a 3-year follow-up. Results: Baseline 7-day-Holter revealed SV runs in 36% of patients and AF in 9% of cases. During a 3-year follow-up, 8 additional cases were confirmed, both in standard care and in repeated Holter (a total of 19% of AF cases). There was no difference with regard to CHADS2VASc score (3.6 ± 1.1 vs. 3.4 ± 1.5; *p* = NS) and left atrium parameters between patients with SV runs and the non-arrhythmic group. Patients with SV runs had a higher incidence of AF both after stroke and in a 3-year follow-up (46% vs. 4%, RR 11.6, *p* < 0.001). In 8 cases, patent foramen ovale was detected during follow-up. Conclusions: A strategy of baseline 7-day-Holter monitoring after stroke allows for disclosing SV runs in every third case and AF in 9% of stroke survivors. Patients with SV runs have a higher incidence of AF (RR 11.6, *p* < 0.001) and should be considered for extended continuous ECG monitoring.

## 1. Introduction

The cause of an acute stroke remains unknown in 20–40% of cases [1]. As it has been recently proved, in many patients with cryptogenic ischemic stroke (CIS), the cause is attributable to undiagnosed, clinically silent atrial fibrillation (AF) [2,3]. Atrial fibrillation is a common arrhythmia, particularly in older age, and although the risk factors for AF are well defined, there are still no reliable tools that would predict the occurrence of AF in a particular patient with the accuracy that would justify taking preventive steps.

The prevalence of AF depends on the population studied and is strictly related to the intensity of the detection strategy applied [4]. In cryptogenic stroke patients, AF can be found in 10% to more than 25% of cases, depending on the timing, duration, and method of monitoring [5], and in many cases, the arrhythmia is asymptomatic and likely to be undetected [6]. Considering that AF is a leading preventable cause of recurrent stroke, active and prolonged screening for atrial fibrillation is crucial for further therapy and prognosis after cryptogenic stroke.

The most reliable data on AF prevalence in stroke survivors come from studies in patients with cardiac implantable electric devices (CIED) [7] and studies utilizing implantable loop recorders (ILR) [8]. While cardiac implantable electric devices (CIED) are a source of valuable data, they are not a diagnostic option in the general population, and may not reflect the incidence of AF in the general population, as the CIED patients have underlying conditions (sinus node dysfunction, heart failure) that make them more likely to have AF. Implantable loop recorders are good diagnostic tools, but the cost and the procedure of implantation make these tools less acceptable for both patients and healthcare systems. Less sensitive but more available and affordable options include: extended Holter monitoring, continuous electrocardiogram (ECG) recording with telemetry, loop recorders, and patient activated (non-continuous) repeated ambulatory ECG recorders.

Despite recent ESC guidelines [9] recommending continuous 24 h ECG monitoring followed by at least 72 h ECG recording, a 12-lead ECG and 24 h Holter monitoring are still routine screening tools for AF screening in stroke survivors. A 7-day non-invasive ECG continuous monitoring is commonly available and despite some drawbacks, acceptable for patients, particularly after the stroke of unknown cause, when patients are more determined to receive appropriate diagnosis and treatment. As we reported in our previous paper [10], in stroke survivors with no arrhythmic findings in 24 h Holter, 7-day Holter monitoring revealed AF in up to 10% of cases and performs better than the guideline-recommended minimum: a 72 h monitoring.

Extended Holter monitoring also provides additional information useful for risk stratification for AF.

SV runs, episodes of non-sustained supraventricular tachycardia (SVT), defined as a sequence of at least 5 QRS, but less than 30 s duration, are thought to predispose to or coincide with AF [11,12]. As Weber-Krüger M et al. [13] reported, patients who had SV runs after stroke were more likely to have a recurrent stroke and novel AF in 3-year follow-up than those without SV arrhythmia.

The study aimed to evaluate the diagnostic value of 7-day-Holter monitoring (as baseline diagnostic and follow-up tool) in CIS patients in whom 24 h Holter was negative for arrhythmia, and the effect of SV runs on the incidence of AF in a 3-year follow-up of the CIS cohort.

## 2. Patients and Methods

We analyzed 78 patients (aged 60 ± 9 years, 45 males) with ischemic stroke with no significant disability and cognitive impairment. In all patients, carotid artery stenosis and any relevant arrhythmic findings in 24-h-Holter (i.e., atrial fibrillation and supraventricular runs ≥5 QRS) were excluded. Patients with hemorrhagic stroke were excluded.

The patients were also screened for other common non-arrhythmic causes of cardiac thromboembolism; however, transesophageal echo was performed during follow-up, not during stroke hospitalization.

Patients were enrolled in the study 4–7 days after admission to the stroke unit. In all patients, we performed the echocardiographic study, and parameters of the left atrium (LA diameter, area, and volume) were recorded. Patients had 7-day-Holter (Lifecard CF/Pathfinder SL, Reynolds Medical, Snoqualmie, WA, USA) monitoring. We recorded AF episodes (duration > 30 s), supraventricular tachycardia (SVT; runs of ≥5 QRS), and other significant rhythm abnormalities in the 7-day recording.

**Figure 1:**
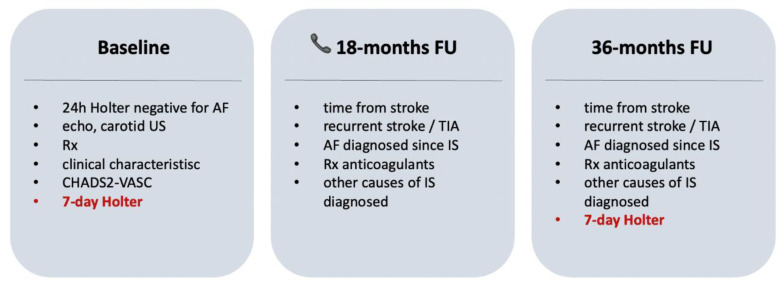
Study design plot.

Patients were followed at 18 and 36 months after stroke. The data gathered during the follow-up period included recurrent stroke or TIA, overt AF diagnosed since stroke (confirmed in ECG), and treatment with anticoagulants. At 18 months, clinical data were gathered during a phone call, while the 36-month visit included data gathering and 7-day Holter (Figure 1).

### Statistical Analysis

Statistical analysis was performed with Statistica 13.1 (Dell). All values were expressed as average (SD). Differences were considered to be significant at *p* < 0.05. To check the normality of the distribution, the Shapiro–Wilk test was performed. In case of a normal distribution, the Student *t*-test was performed, otherwise, the Mann–Whitney U test was used. Qualitative parameters were compared using Pearson’s chi-square and McNemar’s test. The study was accepted by the Ethics Committee of the Medical University of Silesia. The investigation is in line with the principles outlined in the Declaration of Helsinki.

## 3. Results

The baseline characteristics of the studied group are presented in Table 1.

Baseline 7-day-Holter revealed SV runs (≥5 QRS) in 28 patients (36%). SVT duration was 5–164 beats; rate 120–188 bpm. In 7 out of 28 SVT patients, AF episodes (duration 40 s–7 min) were also found. All cases with confirmed AF had also at least one episode of SV run in the same recording. Besides, 27 patients (35%) presented bradycardia (<50 beats per minute), and in one case, pacemaker implantation was necessary three weeks after the stroke-related hospitalization for re-confirmed in another Holter symptomatic brady–tachy syndrome.

Follow-up data are shown in Table 2. During the first 18 months after stroke, 3 patients were diagnosed with overt atrial fibrillation. Besides, PFO was confirmed in seven cases and thrombophilia in one case. Nine patients were lost to follow-up. During the subsequent 18 months, another 2 patients had a clinical diagnosis of AF confirmed and there was one more case of PFO diagnosed. Additionally, two cases were lost to follow-up. There were no recurrent strokes reported in the cohort throughout the observation period. All patients in whom AF had been confirmed received oral anticoagulant including the three patients in whom the final Holter revealed AF.

Based on the initial 7-day-Holter results, patients were classified into two groups: patients with SV runs and patients without SV arrhythmia. As seen in Table 3, the groups were similar with regard to age, CHADS2-VASC score, and left atrium parameters, but patients with SV arrhythmia in the initial Holter had a significantly higher incidence of AF confirmed in the baseline Holter or throughout the follow-up period (Table 4).

Finally, we compared the AF patients vs. those without AF (lost to follow-up patients were excluded) (Table 5). Out of 15 patients with confirmed AF, 13 (87%) had had SV runs in a baseline 7d recording. SV runs were present in 14 out of 52 (27%) patients without confirmed AF and in 1 out of 11 (9%) LTFU cases.

In our cohort, patients with AF were on average eight years older, had higher CHADS2VASc score, and their LA parameters were not significantly different from non-AF patients. SV runs are more than three times more common in peri-stroke 7-day Holter of patients with AF in 3-year follow-up periods.

## 4. Discussion

Over the past decade, the strategy to search for AF in stroke survivors has evolved from a standard ECG and a 24 h ECG monitoring to the current guideline-recommended extended monitoring, which involves at least 72 h Holter, but also telemonitoring or implantable loop recorders in selected cases. The everyday clinical practice, however, commonly utilizes 24-h-Holter. This is both because of limited access to extended monitoring technologies and the lack of tools for selecting the right population for these modalities. In a real-world setting, there is no reasonable tool for non-invasive and long-term monitoring for AF. The advantages and disadvantages of available monitoring strategies are summarized in Table 6.

The recent utilization of smartwatches (particularly those with an ECG capability) seems to be a promising technology, yet not without limitations. In the most popular available systems, AF is detected based on photoplethysmographic signals, and it requires the patient’s action to record the ECG strip (if such an option is possible). Besides the multiple, false alerts caused by irregular rhythm, forcing patients to record an ECG may cause more confusion than benefits. However, in selected, highly motivated groups, this technology may shorten the time to diagnosis.

The 24 h Holter yields a detection rate of 1–2% in the stroke survivor group. From the design of our study, it is also clear that 24 h recording is not a satisfactory strategy. A report by Jabaudon et al. revealed AF in 5% using 24 h-Holter, then in another 5.7% using 7-day recording [14]. Wachter and co-workers identified AF in 16% of CIS survivors using a strategy of repeated 10-day Holter (3 rounds within 6 months follow up) vs. standard care [15]. Recently, to make long-term monitoring more acceptable for patients, wearable or patch recorders are being more popular. In a study utilizing ECG patch (ZioPatch, iRhythm) a detection rate for AF in patients with a recent history of stroke was 5%, with a median recording duration of 13 days [16]. In our study, single 7-day recording performed just after stroke identified 7 cases (9%) of AF and another 7-day Holter at the end of a 3-year observation—additional 3 cases of AF (a total of 10/78 cases, 13%).

The strategy of repeated 7-day Holter is inferior to continuous monitoring strategies. Implantable loop recorders are a golden standard in searching for atrial fibrillation. In the CRYSTAL-AF study (Reveal XT ICM, Medtronic, in cryptogenic stroke patients), the rates of AF detection were 8.9% after 6 months, 12.4% after 12 months, and 30% after 36 months. Importantly, the median time to AF detection was 41 days during the first 6 months of follow-up and went up to 84 days when follow-up was extended to 3 years [8]. This may put in doubt strategies that focus on shorter monitoring duration. On the other hand, in the EMBRACE study, the AF detection rate for 30 days external loop recorder was 16.1% in post-stroke patients [11].

Data from pacemaker and ICD patients suggests that the incidence of AHRE (atrial high rate episode) in patients after stroke is 30–45% in 30 months of follow-up. However, as stated before, data from CIED patients cannot be directly translated to the general population. Moreover, the ASSERT and TRENDS studies suggest that the duration of AF episodes and AF burden affect thromboembolic risk, particularly in patients with low clinical risk factor profile [7,17].

The cost of implantable monitoring devices and invasive procedure of implantation raise two questions: whether commonly used, inexpensive, and non-invasive forms of monitoring can be sufficient, and how to select the patients for long-term monitoring (i.e., ILRs).

As we report, the patients in whom AF was confirmed over a 3-year follow-up did not differ much from those without proved AF with regard to commonly recognized risk factors (i.e., factors included in CHADS-VASc scale, LA parameters). They did, however, differ with regard to the incidence of SV runs—SV arrhythmia thought to coincide with and/or predispose to AF. We searched for SV runs in the baseline 7-day recording, and found a significant relation between SV runs and AF revealed at any time during 3-year follow-up. In the analysis of patients from the prospective FindAF trial [13], patients with stroke who had SV runs were more likely to have a recurrent stroke and showed 2.5 times more novel AF in 3-year follow-up than the controls. In our study, recurrent strokes were not observed in similar FU time, but the baseline SV runs were clearly related with AF revealed immediately after stroke or during FU.

In summary, it is equivocal that any type of extended ECG monitoring is significantly better than a standard 24 h-Holter. Repeated 7-day Holter in stroke survivors reveals more novel AF cases, also late on a follow-up route. Moreover, SV runs detected in the initial 7-day monitoring is related to an increased incidence of AF and may be a useful tool to select candidates for closer ECG follow-up.

## 5. Limitation

One limitation of the study is a lack of a reference method (preferably implantable cardiac monitor) to assess the real incidence of AF and its burden. Another limitation is the number of participants.

## 6. Conclusions

A strategy of baseline 7-day Holter after stroke not only allows for disclosing AF in 9% of stroke survivors, but also reveals SV runs in every third patient after stroke. Patients with SV runs have a higher incidence of AF both early after stroke and in 3-year follow-up and should be considered for extended continuous ECG monitoring.

## Figures and Tables

**Table 1:** Baseline characteristics of the studied group.

Parameter	
Age (years), mean ± SD	60.3 ± 9.1
CHADS2-VASC score, mean ± SD	3.60 ± 1.20
Congestive Heart Failure, n (n%)	3 (4%)
Ejection Fraction (%), mean ± SD	59 ± 4
Mitral regurgitation mild/moderate/severe	31 (40%)/8 (10%)/0 (0%)
Hypertension, n (n%)	40 (51%)
Diabetes, n (n%)	10 (13%)
Vascular Disease	5 (6%)
Beta-blocker before stroke	28 (36%)
Antiplatelet at discharge—ASA	62 (79.5%)
Antiplatelet at discharge—clopidogrel	16 (20.5%)
Oral anticoagulants at discharge	0 (0%)

Numerical data presented as mean ± SD (standard deviation) or n (n%).

**Table 2:** The 36 months of follow-up.

	Baseline (*n* = 78)	FU—18 Months	FU—36 Months	Total
Time from stroke (months, median)	N/A	17 (14–23)	36 (33–44)	--
AF in 7d Holter	7	N/A	+3	10 (12%)
Overt AF Dx after stroke	N/A	3	+2	5 (6%)
Total with confirmed AF	7	3	5	15 (19%)
Other causes of CIS—PFO	0	+7	+1	8 (10%)
Other causes of CIS—thrombophilia	0	+1	0	1
Recurrent stroke	N/A	0	0	0
Lost to follow up	N/A	9	+2	11 (14%)

**Table 3:** Patients with SV runs vs. without SV runs—clinical and echo parameters.

	SV Runs (*n* = 28)	No SV Runs (*n* = 50)	*p*
Age (years)	61 ± 10	59 ± 9	NS
CHADS2-VASC score	3.6 ± 1.1	3.4 ± 1.5	NS
Ejection fraction (EF) (%)	58 ± 5	59 ± 6	NS
Left atrium diameter (mm)	37 ± 9	38 ± 6	NS
Left atrium volume (mL)	58 ± 17	62 ± 26	NS

**Table 4:** Comparison of the incidence of AF in patients with and without SV runs.

	Total *n* (%) *n* = 78	SV Runs *n* (%) *n* = 28	No SV Runs *n* (%) *n* = 50	RR	95% CI	*p* *
AF	15 (19%)	13 (46%)	2 (4%)	11.6	2.82–47.4	0.0007

* two-tailed Pearson’s Chi-square test.

**Table 5:** Patients with vs. without atrial fibrillation—clinical and echo parameters.

	AF (*n* = 15)	No AF (*n* = 52; Per Protocol)	*p*
Age (years)	66 ± 8	58 ± 10	*p* < 0.05
CHADS2-VASC score	3.9 ± 1.1	3.5 ± 1.2	*p* = 0.57
Ejection fraction (EF) (%)	57 ± 4	59 ± 5	NS
Left atrium diameter (mm)	39 ± 7	35 ± 6	NS
Left atrium volume (mL)	64 ± 17	58 ± 26	NS
SV runs in baseline Holter	13 (87%)	14 (27%)	<0.001

**Table 6:** Advantages and disadvantages of ECG monitoring strategies for detection of atrial fibrillation.

ECG Monitoring Technology	Advantages	Disadvantages
24–72h Holter	low cost	likely to miss AF
7-day Holter	low cost, longer recording	inconvenient
patch recorder	7–14 days recording, better adherence	recording length similar to Holter, higher cost, limited leads
patient monitors–intermittent short ECG recordings	low cost, convenient (no wires)	likely to miss asymptomatic, short-lasting arrhythmias, requires patients action
pulse-based AF detection (smartwatch, bands)	convenient, affordable	a high proportion of false alerts needs ECG confirmation
implantable cardiac monitors (ICM)	long term monitoring (years)	an invasive procedure, high cost

## Data Availability

The data presented in this study are available on request from the corresponding author.
